# Acute Cryptococcal Meningitis in a Patient With Idiopathic CD4 Lymphocytopenia: A Rare Clinical Entity

**DOI:** 10.7759/cureus.43417

**Published:** 2023-08-13

**Authors:** Vaishnavi Kavirayani, Arundhati Negi, M. Mukhyaprana Prabhu

**Affiliations:** 1 Internal Medicine, Kasturba Medical College, Manipal, IND

**Keywords:** opportunistic infections, lumbar puncture, immunocompetent host, idiopathic cd4 lymphocytopenia, cryptococcal meningitis

## Abstract

Cryptococcal meningitis is a known cause of opportunistic infection in immunocompromised patients, especially those with AIDS. Very few cases exist in literature where cryptococcal meningitis is seen in patients without evidence of HIV infection. Here, we describe a case of an elderly woman presenting with clinical features of meningitis.

Our patient tested positive for cryptococcal antigen (CRAg) in the CSF and growth of *Cryptococcus neoformans *was obtained in CSF culture. Further laboratory investigations revealed CD4 lymphocytopenia (233 cells/μl) in the absence of HIV infection. When we checked the CD4 count, beyond a period of six weeks, it was reported to be low, which confirmed our diagnosis of idiopathic CD4 lymphocytopenia (ICL). She was successfully treated with amphotericin B along with flucytosine for two weeks and discharged on maintenance antifungal therapy for eight weeks. This case emphasizes the need to maintain a high index of suspicion and consider the possibility of opportunistic infections even in the absence of HIV infection for timely diagnosis and treatment.

## Introduction

*Cryptococcus neoformans* is primarily an opportunistic pathogen that causes life-threatening infections in immunocompromised hosts, especially patients with T-cell deficiencies including severe combined immunodeficiency syndrome (SCIDS) or AIDS. It enters through the respiratory or nasal route and then spreads via blood to extrapulmonary tissues, mainly the brain and CSF, leading to cryptococcal meningitis.

The occurrence of cryptococcal meningitis in patients with idiopathic CD4 lymphocytopenia (ICL) is a relatively new and rare occurrence. ICL is defined as a clinical state in individuals with depleted circulating CD4 T lymphocyte count (<20% of total T cells or <300 cells/μl) at two different points in time, a minimum of six weeks away, with no laboratory evidence of infection with HIV-1 or HIV-2, and the absence of any specific immunodeficiency or treatment associated with decreased levels of CD4 T lymphocytes [1]. Current literature shows few cases of ICL that are associated with various opportunistic infections like tuberculosis, parasitic, viral, and fungal infections, also present in individuals who are HIV-positive [2]. The etiology, pathogenesis, clinical features, and optimal treatment options remain unclear.

## Case presentation

An elderly female with a known history of hypertension and hypothyroidism presented with high-grade fever, vomiting, and bifrontal headache of throbbing nature of four days duration. There was no history of abdominal pain, loose stools, giddiness, blurring of vision, deafness, or loss of consciousness. Our patient denied any history of recent travel or sick contact. On physical examination, she was normotensive with a blood pressure of 120/80 mm Hg, a pulse rate of 100 beats/min, and a temperature of 100.2°F. CNS examination revealed neck stiffness and Kernig’s sign was positive. Fundoscopy revealed papilledema. She was mildly disoriented. The remaining neurological examination was unremarkable.

**Figure 1:**
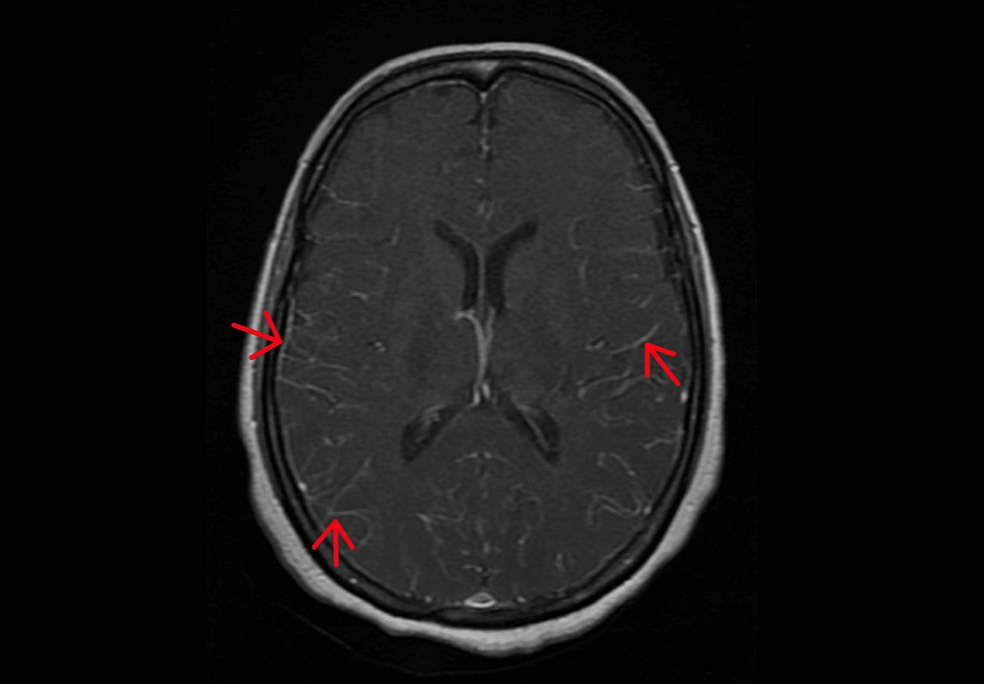
MRI brain showing meningeal enhancement

Laboratory investigations showed leukocytosis (11.8 x 10^8^/μl) with neutrophil predominance (79.4%), thyroid-stimulating hormone (TSH) levels of 1.480 ulU/ml, and elevated erythrocyte sedimentation rate (ESR) (83 mm/h). Antibodies to HIV, Western blot, and HIV viral load tested negative. CSF sample obtained tested negative for herpes simplex virus (HSV), varicella-zoster virus (VZV), Japanese encephalitis, hepatitis B and C, tuberculosis, dengue, leptospirosis, and scrub typhus. MRI study of the brain showed meningeal enhancement (Figure 1). A diagnosis of cryptococcal meningitis was arrived at based on the CSF findings of elevated opening pressure of 250 cm H_2_O, hypoglycorrhagia, leukocytosis (white blood cells [WBCs] 300 with 59% lymphocytes), and high protein content (309 mg/dl) (Table 1). India-ink staining of CSF sample was positive for *Cryptococcus neoformans* and CSF culture yielded *Cryptococcus neoformans* as well (S 0.5 for amphotericin B). Cryptococcal antigen (CRAg) was found in serum and CSF. CSF serum glucose ratio was 0.21. The patient was not on any immunosuppressive therapy. Owing to high suspicion, further investigations were carried out, which revealed CD4 lymphocytopenia (233 cells/μl).

**Table 1:** CSF analysis CSF: cerebrospinal fluid.

CSF characteristics	Value (on admission)
Appearance	Clear
Opening pressure (cm H_2_O)	250
Glucose (mg/dl)	25
Protein (mg/dl)	309
Leucocytes (cells/mm^3^)	300
Neutrophils (%)	41
Lymphocytes (%)	59 (reactive form (+))
CSF/serum glucose ratio	0.21

We started her on a two-week course of intravenous amphotericin-B infusion (4 mg/kg) along with flucytosine (25 mg/kg every 6 hours). Repeated lumbar puncture was performed as part of management. CSF analysis after two weeks showed persistence of CRAg and positive India-ink staining for Cryptococcus. The sample was again sent for fungal culture and was reported sterile after five days of incubation. Repeat CSF analysis showed CRAg positivity, but cryptococcal organisms were not seen on India-ink staining. Repeat culture after one week again reported negative. As the patient became symptomatically better and culture was sterile, she was started on maintenance therapy of oral fluconazole for cryptococcal meningitis, for eight weeks. On subsequent follow-ups after six months and one year, respectively, the patient does not complain of any focal neurological deficits, altered behavior, seizures, or loss of vision. Cell counts revealed low CD4 levels, hence establishing our diagnosis of ICL.

## Discussion

Cryptococcal meningitis is a severe and uncommon disease generally affecting immunocompromised individuals. It is a rare cause of infection in immunocompetent hosts, especially in the central nervous system. Following an increase in cases of opportunistic infections like cryptococcosis in individuals without known disease with low CD4 cell count, the term idiopathic CD4 lymphocytopenia was first coined in 1992 by the Centers for Disease Control and Prevention (CDC) [1]. ICL in immunocompetent individuals is extremely rare. Transient CD4 lymphocytopenia where low CD4 count may also be a result of low total WBC count is relatively more common and seen in 0.4-4.1% of healthy HIV seronegative individuals [3]. In a large study involving 47 patients with ICL, it occurred more commonly in males (M: F ratio of 1.6:1) and was found mostly among the age group of 17-78 years [4]. Very few cases with familial history exist in the current literature [5].

ICL has no known etiology. Recently, the focus of the study has shifted to various aspects of the immune system. This condition is increasingly predisposed to the occurrence of opportunistic infections, typically including mycobacterial, cryptococcal, viral pathologies, malignancies, and autoimmune diseases [6]. In a study conducted by Yarmohammadi, et al., 71% of patients had opportunistic infections, 17% had malignancies, and 13% had unexplained neurological problems [7]. Currently, it is postulated that these conditions are a result of dysregulation of the immune system, associated with the development of antibodies to T-cells [8]. Another hypothesis also suggests increased T-cell apoptosis, decreased precursors of T-cells, or decreased cytokine production [9].

Our case is one of the few reported in India. Even among the cases reported, the most common associated diseases were tuberculosis and cryptococcal meningitis. Two cases of dermal candidiasis in the setting of ICL have also been reported by Mukherjee, et al. [10].

*Cryptococcus neoformans* infection mainly occurs in an immunocompromised host. Its association with ICL in an immunocompetent individual is extremely rare, due to which no specific guidelines for prophylaxis and treatment have been outlined. Management is usually along the same lines as seen in HIV-infected individuals with opportunistic diseases. It is thought to be rational to continue voriconazole or fluconazole life-long in individuals with CD4 lymphocytopenia, who tested negative for HIV infection [2]. Further, treatment with IL-2 (interleukin-2) has been suggested for enhancing CD4 cell counts.

## Conclusions

The scientific community must aim to bridge the existing gaps in knowledge about the etiology, pathogenesis, and optimal treatment modalities of ICL. It will thus enable us to set standardized recommendations and treatment guidelines and address the current challenge posed to physicians and modern medicine. Our case report highlights the importance of approaching cases without bias and maintaining a high index of suspicion, for timely diagnosis and optimal treatment of any rare disease. Immunodeficiency is not necessarily associated with HIV and testing for other opportunistic infections should still be carried out. In such patients who develop opportunistic infections in the absence of HIV infection or other associated immunodeficiencies, the possibility of ICL must therefore be considered.
